# Facile One-Pot Method for All Aqueous Green Formation of Biocompatible Silk Fibroin-Poly(Ethylene Oxide) Fibers for Use in Tissue Engineering

**DOI:** 10.1021/acsbiomaterials.1c01555

**Published:** 2022-03-01

**Authors:** Phoebe Louiseanne Heseltine, Cem Bayram, Merve Gultekinoglu, Shervanthi Homer-Vanniasinkam, Kezban Ulubayram, Mohan Edirisinghe

**Affiliations:** † Department of Mechanical Engineering, University College London, Torrington Place, London WC1E 7JE, United Kingdom; ‡ Institute of Science and Technology, Department of Nanotechnology and Nanomedicine, Hacettepe University, Ankara 06800, Turkey; § Faculty of Pharmacy, Department of Basic Pharmaceutical Sciences, Hacettepe University, Ankara 06800, Turkey

**Keywords:** pressurized gyration, silk fibroin, PEO, aqueous, biocompatible, bone tissue, green chemistry

## Abstract

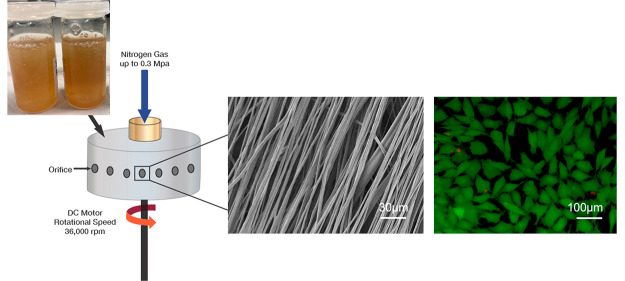

Silk fibroin (SF) fibers are highly regarded in tissue engineering because of their outstanding biocompatibility and tunable properties. A challenge remains in overcoming the trade-off between functioning and biocompatible fibers and the use of cytotoxic, environmentally harmful organic solvents in their processing and formation. The aim of this research was to produce biocompatible SF fibers without the use of cytotoxic solvents, via pressurized gyration (PG). Aqueous SF was blended with poly(ethylene oxide) (PEO) in ratios of 80:20 (labeled SF-PEO 80:20) and 90:10 (labeled SF-PEO 90:10) and spun into fibers using PG, assisted by a range of applied pressures and heat. Pure PEO (labeled PEO-Aq) and SF solubilized in hexafluoro-isopropanol (HFIP) (labeled SF-HFIP) and aqueous SF (labeled SF-Aq) were also prepared for comparison. The resulting fibers were characterized using SEM, TGA, and FTIR. Their in vitro cell behavior was analyzed using a Live/Dead assay and cell proliferation studies with the SaOS-2 human bone osteosarcoma cell line (ATCC, HTB-85) and human fetal osteoblast cells (hFob) (ATCC, CRL-11372) in 2D culture conditions. Fibers in the micrometer range were successfully produced using SF-PEO blends, SF-HFIP, and PEO-Aq. The fiber thickness ranged from 0.71 ± 0.17 μm for fibers produced using SF-PEO 90:10 with no applied pressure to 2.10 ± 0.78 μm for fibers produced using SF-PEO 80:10 with 0.3 MPa applied pressure. FTIR confirmed the presence of SF via amide I and amide II bands in the blend fibers because of a change in structural conformation. No difference was observed in thermogravimetric properties among varying pressures and no significant difference in fiber diameters for pressures. SaOS-2 cells and hFOb cell studies demonstrated higher cell densities and greater live cells on SF-PEO blends when compared to SF-HFIP. This research demonstrates a scalable and green method of producing SF-based constructs for use in bone-tissue engineering applications.

## 1 Introduction

Silk fibroin (SF) fibers offer many advantages for biomaterial scaffolds in tissue engineering because of their unique, highly tailorable physicochemical and mechanical properties.^1^ Silk derived from the domesticated *Bombyx mori* (*B. mori*) silkworm possesses good biocompatibility, oxygen and water permeability, a broad range of mechanical properties, and biodegradability. Because of the established history of *B. mori* species in the textile trade its cocoon production is in large supply, it is also renewable, recyclable, and biodegradable.^2,3^ As such, it has been utilized in a number of tissue scaffolding applications such as in the cornea, skin, bone, cardiac patches, and periodontics, as well as drug delivery and, lately, more broadly in medicine, in the field of bioelectronics.^4−11^

Scaling SF fiber production for tissue engineering applications is challenging. Silk cocoons demand batch processing, which introduces variability in polymer properties among stocks, such as crystallization degree, molecular weight, and amount of degumming.^12^ This can have a knock-on effect on mechanical performance, biocompatibility, and degradation of regenerated fibers in vivo.^13−15^ Additionally, the use of chaotropic solvents in the dissolution of SF is problematic because of their environmental impact and toxic effect on cells.^16^ A broad range of methods are established in generating SF fibers—these typically fall into the categories of wet spinning, electrospinning, or dry spinning.^17^ Wet spinning methods can induce desirable properties such as low diameter fibers and high strength but can have negative environmental impact because of the requirement of a solvent-based coagulation bath to assist in fiber drying.^18^

Electrospinning has successfully produced pure SF fibers and polymer hybrid SF fibers via both water and organic solvent-based solutions, offering the advantages of nanoscale fiber production to replicate the extracellular matrix.^19,20^ Fibers formed from aqueous SF solution offer an attractive alternative to many of the chaotropic solvents (i.e., formic acid or hexafluoro-isopropanol (HFIP)) that are currently used. Kishimoto et al. generated aqueous SF fibers at low concentration via the electrospinning of high-molecular-weight SF with alkaline pH to induce fiber formation.^21^ The scalability of ES is hampered, however, by low production rates; additionally, the application of an electric field can lead to tight packing of fibers, which can negatively affect cell infiltration.^22^ Combined with the inherent reproducibility issues that silk possesses, the scalability of ES for SF biomaterial fiber fabrication is limited.

Centrifugal spinning is a dry spinning strategy that utilizes a rotating spinning head to eject fibers. It is capable of producing fibers at speeds order of magnitudes higher than ES and at lower cost.^23^ Liu et al. successfully fabricated SF fibers solubilized in formic acid using a centrifugal spinning method.^24^ Typically, their centrifugal spinning method produced broader fiber diameters than ES but more loosely packed mats, although no cell compatibility studies were performed.

Biomimetic approaches that replicate the spinning process occurring within the native silk gland have also shown some success in generating fibers with good mechanical properties. Strategies have involved tuning the pH and salt concentration of spinning solutions, as well as using novel solution shearing strategies, such as in the work of Luo et al., who used a microfluidic chip to replicate the geometry of the silk gland.^25,26^ The majority of these studies suffer from low production rates, often producing only a single SF thread at a time.

Pressurized gyration (PG) is a high-throughput dry spinning method that employs the use of centrifugal spinning and solution blowing to rapidly produce fibers with tunable properties, because of the ability to vary applied gas pressure to the solution. Earlier proof-of-principle work by the authors demonstrated for the first time that it was possible to generate SF fibers using the PG method, with hexafluoroisopropanol solvent.^27^ However, HFIP is difficult to remove from the fibers for cell compatibility and is prohibitively expensive.

The aim of this research is 2-fold: (1) to demonstrate the feasibility of producing environmentally friendly, solvent-free, SF-based fibers using PG, and (2) to evaluate their biological performance against SF-fibers produced via the more commonly used organic solvent, HFIP. PEO is highly biocompatible and elastic and is also used in blending, having demonstrated previous success in improving the processability of the spinning solution, as reported with E-spin methods.^28^ It has also been spun successfully in aqueous form using PG.^29^ Herein, SF-PEO fibers are spun in an aqueous-based solvent system for the first-time using PG. The fibers are characterized, and their cytotoxicity is evaluated using SaOS-2 and hFob cells.

## 2 Experimental Section

### 2.1 Materials

30% degummed *B. mori* SF was purchased from CareSilk (Lecce, Italy). Slide-a-Lyzer dialysis cassettes of 3500 MWCO were purchased from ThermoFisher Scientific (Loughborough, UK). Lithium bromide, Poly(ethylene oxide) Mw 2 × 10^5^ g mol^–1^ and 1,1,1,3,3,3-Hexafluor-2-propanol (HFIP) were purchased from Sigma-Aldrich (Gillingham, UK). Ultrapure water was obtained using a Millipore filter.

### 2.2 Fabrication of Silk Blend Fibers

#### Preparation of Regenerated SF

*B. mori* SF was prepared using a modified protocol originally outlined by Rockwood et al.^30^ 30% degummed SF was dissolved in 9.3 M LiBr solution at 70 °C for 4 h. This solution was dialyzed against ultrapure water using a Slide-a-Lyzer dialysis cassette (MWCO 3500) to produce aqueous SF at a concentration of 3 w/v%. SF was centrifuged at 9000 rpm for 15 min at 25 °C to remove aggregates that occurred due to dialysis. Silk was then concentrated further through evaporation in an oven at 30 °C for 72 h. The final concentration of aqueous silk solution was 50 w/v%, as determined by weighing the remaining solid after drying. SF was dissolved directly in HFIP for the comparative samples at a concentration of 8 w/v%, chosen based on previous results.^27^

#### Preparation of Spinning Solutions and Solution Rheology

Aqueous 50 w/v% Mw 2 × 10^5^ g mol^–1^ PEO solutions were prepared by fully dissolving the solid in deionized water at a concentration of 15 w/v%. SF solutions and 15 w/v% PEO solution were blended at ratios of 80:20 (SF-PEO) and 90:10 (SF-PEO) in preparation for spinning. All solutions were magnetically stirred for 24 h prior to spinning at room temperature. These are summarized in Table 1 and each solution is referred to by its name as described in the table.

**Table 1:** Polymer Spinning Solution Compositions

name	silk fibroin (w/v%)	PEO (w/v%)	solvent
SF-PEO 80:20	40	3	deionized water
SF-PEO 90:10	45	1.5	deionized water
PEO-Aq		15	deionized water
SF-HFIP	8		HFIP
SF-Aq	50		deionized water

The surface tensions of the spinning solutions were characterized using the Wilhelmy plate method (Tensiometer K9, Kruss GmbH, Germany) and repeated five times to calculate the mean (Table 2). The viscosities of all the solutions were measured using a Brookfield DV-111 rotational viscometer (Harlow, UK) with spindle size 18, at a shear stress of 3.5 Pa. 6.7 mL samples were loaded of each solution into the rheometer, repeated viscosity measurements were taken as shear rate was increased, at a torque of above 70%. All measurements were taken in the temperature range of 24–25 °C. The recorded viscosities in Table 2 are the mean viscosity over 10 measurements across increasing shear rate.

**Table 2:** Surface Tension and Viscosity Values of Prepared Solutions for Spinning

	surface tension (Wilhelmy plate method) (nM/N)	rotational viscosity (cP)
PEO-Aq	64.8 ± 0.6	360.8 ± 3.7
SF-PEO 80:20	54.3 ± 0.5	29.9 ± 0.4
SF-PEO 90:10	52.5 ± 1.7	26.1 ± 0.2
SF-HFIP	34.4 ± 1.3	17.6 ± 0.6
SF-Aq	45.8 ± 0.2	19.3 ± 0.2

#### Fiber Formation Using Pressurized Gyration

**Figure 1:**
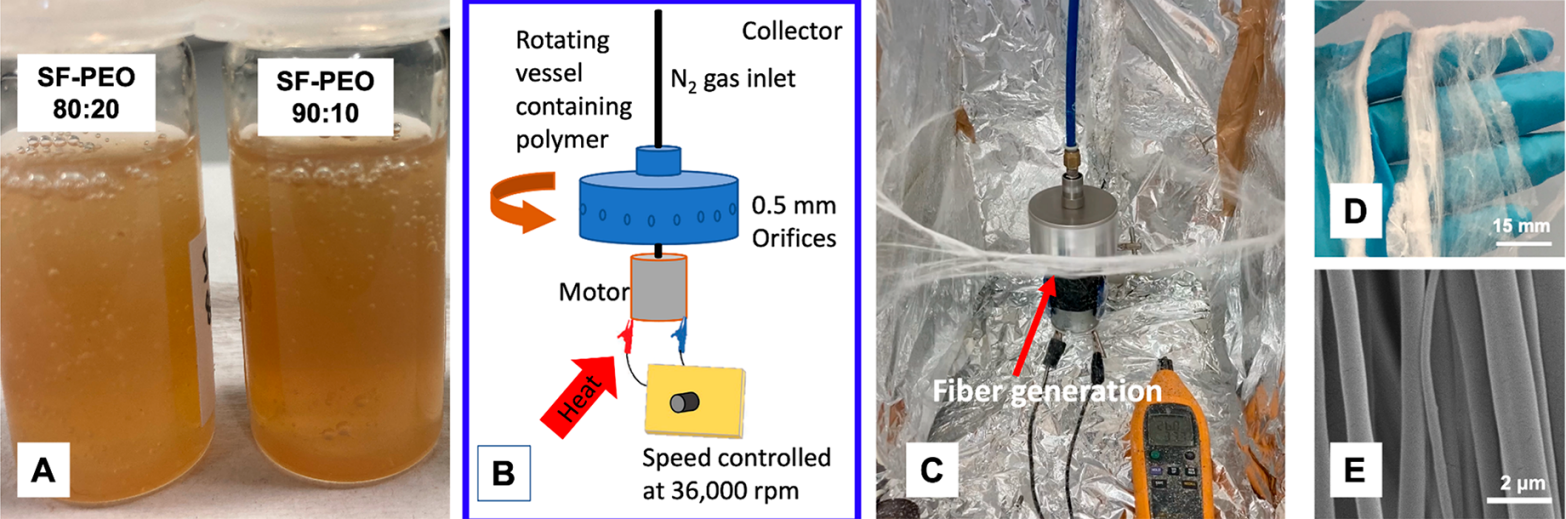
Regenerated silk fibroin fabrication using PG: (A) aqueous silk blends, (B) PG setup for polymer solutions, (C) fiber bundle generation inside the PG chamber after 5 s, (D) macroscale image of 1 mL of spun SF-PEO 80:20 fibers produced at a 0.2 MPa applied pressure and 36 000 rpm, (E) corresponding SEM image of the fiber sample.

Fibers were created using a custom-built setup detailed in Figure 1. In the PG system, a rotating aluminum cylindrical vessel (60 mm in diameter ×35 mm high) was used with 24 perforations surrounding the core, with each orifice having an internal diameter of 0.5 mm. The device radius was 30 mm with a wall thickness of 1 mm. For each of the solutions, 1 mL was loaded into the vessel, with the vessel lid screwed shut. Each of the solutions were spun at an applied pressure of 0.1, 0.2, and 0.3 MPa, all at an apparent speed of 36 000 rpm and at a collection distance of 120 mm, for approximately 30 s. A heat gun reaching 80 °C was applied subsequently to rotation and gas pressure to assist in fiber drying and fibers were recovered from the walls of the collector once deposited onto aluminum sheeting. The ambient temperature was 19.1–23.2 °C. The relative humidity was 28.2–44.3%.

### 2.3 Scanning Electron Microscopy (SEM)

Fiber morphology and fiber diameter distributions were evaluated by scanning electron microscopy (SEM). The samples were sputter coated twice with gold (Q150R ES, Quorum Technologies) for 3 min prior to imaging using SEM (Hitachi S-3400n). The mean fiber diameter distribution was analyzed using *ImageJ* software.

### 2.4 Attenuated Total Reflectance-Fourier Transform Infrared Spectroscopy (ATR-FTIR)

ATR-FTIR spectra were recorded on a Nicolet is50 infrared spectrometer (ThermoFisher Scientific (Loughborough, UK)). Spectra of SF-HFIP, SF-PEO 80:20, SF-PEO 90:10, and PEO-Aq fiber samples were measured in the range of 4000–650 cm^–1^. Thirty-two scans were performed at a resolution of 4 cm^–1^ and collected graphs were superposed to investigate functional differences of the gyrospun polymer fibers in terms of applied pressures and the ratio of the blend solutions.

### 2.5 Thermogravimetric Analysis (TGA)

Thermogravimetric (TGA) analysis was conducted using a thermal analyzer (TA Instruments Q600-SDT, USA) to investigate and compare the thermal stability of SF-HFIP, SF-PEO 80:20, SF-PEO 90:10 and PEO-Aq fiber samples. The analysis was performed under nitrogen gas at a flow rate of 100 mL/min in the temperature range of 25 and 600 °C, heated at 10 °C/min. One to five milligrams of each sample was weighed and sealed in a platinum pan. An empty pan was used for reference.

### 2.6 Cell Studies

#### Live/Dead Assay and Cell Proliferation Assays

Sterilization of gyrospun fiber samples for use in cell culture was carried out by soaking 200 mg of fiber from SF-HFIP, SF-PEO 80:20 and SF-PEO 90:10 samples in 70% ethanol solution prior to UV sterilization at 254 nm, for 20 min inside a laminar flow cabinet. Samples were washed twice with phosphate buffer, dried, and placed into 1 mL of cell culture media (90% DMEM+10% fetal bovine serum supplemented with 2 mM l-glutamine) inside sealed vials for 72 h. Tests were carried out in triplicate for each time interval.

Indirect cytotoxicity of SF-HFIP, SF-PEO 80:20 and SF-PEO 90:10 fibers were evaluated against an SaOS-2 cell line (ATCC, HTB-85TM) and human fetal osteoblast “hFOB” 1.19 (ATCC, CRL-11372) cell lines in 2D culture conditions. The cells were thawed from stock and seeded at a concentration of 1 × 10^5^ cells/mL into a 25 cm^2^ tissue culture plate at 37 °C in a 90% humidified incubator with 5% CO_2_. Samples were incubated overnight with Dulbecco’s modified Eagle’s medium (DMEM) supplemented with 10% fetal bovine serum (FBS), 2.5 mL of l-glutamine, and 20 000 U/mL pen/strep solution. After reaching 80–90% confluency, the cells were harvested from the tissue culture flask using Trypsin EDTA and seeded into 96-well plates at a density of 1 × 10^4^ cells per well. After 24 h, the cell media was replaced with the media interacting with the gyrospun fiber samples and subjected to an additional 24 h of incubation. Cell viabilities were evaluated with a fluorescence based Live/Dead assay kit (Invitrogen, Paisley, UK). After 24 h of incubation with the cell media, the fibers were removed from the wells and 200 μL PBS solution containing 2 mM calcein AM and 4 mM ethidium homodimer-1 was added. Cells interacted with fluorescence dyes in darkness for 30 min and representative images of green (live) and red (dead) cells were taken and merged using ImageJ software.

The cell proliferation assay was conducted at time points of 1, 4, and 7 days via direct seeding of cells onto gyrospun fiber samples in 48 well plates, at a density 10^4^ cells/well. Following the incubation periods, the cell media was replaced with 200 μL of fresh medium containing 20 μL of MTT (3-[4,5-dimethylthiazol-2-yl]-2,5-diphenyltetrazoluim bromide solution (5 g/mL), diluted with DMEM without phenol red) and added to each well. The samples were incubated for a further 4 h in darkness at 37 °C. Formazan crystals were formed due to mitochondrial activity with isopropanol-HCl solution (0.04 M HCl in absolute isopropanol). A 100 μL medium from each well was aspirated and transferred to a 96-well analyzing plate. Relative cell viabilities were estimated against the negative control group using the absorbance spectra at a wavelength of 570 nm.

### 2.7 Statistical Analyses

*ImageJ* software was used to measure fiber diameters in more than 10 SEM images and standard deviation of fiber diameter was measured in 100 fibers. A Shapiro-Walk test was performed on fiber diameter data to assess the normality of sample distribution. A one-way ANOVA test was performed on cell proliferation data with multiple comparisons including a Tukey test. *GraphPad Prism v8.0* was used.

## 3 Results and Discussion

### 3.1 Formation of Fibers via a Pressurized Gyration Spinning Process

Fibers were formed from SF-HFIP, SF-PEO 80:20, SF-PEO 90:10, and PEO-Aq spinning systems using PG. No fibers were formed from SF-Aq solution. Fiber formation occurs due to manipulation of the Rayleigh–Taylor instability of the polymer solution as it emerges from the PG vessel orifices.^29^ The instability between fluids of different densities (polymer solution and air) is apparent when the low-density fluid applies force to the high density fluid. On application of centrifugal force and pressure, a surface tension gradient occurs along the polymer liquid–air interface that separates the droplet from the surrounding air. Mass transfer occurs along the interface due to a surface tension gradient and a flow is induced to the tip of the polymer. Marangoni stress (mass transfer along an interface between two fluids due to a surface tension gradient) occurs as a result of the surface tension gradient generated, inducing a flow to the tip of the polymer droplet.^31^

The rotation of the drum and centrifugal force is the main driver of solution ejection from the orifices on the surface of the drum. Stretching and elongation of the jet occurs because of the effect of applied pressure and rotational force, producing fibers. Heat-assisted solvent drying and evaporation occur until the fibers solidify, enabling them to be collected from the walls of the PG chamber.^32^ Recent experimental and simulation work in our group has shown that this addition of pressure also serves to increase the production yield of the spinning solution as it increases the speed at which the solution is ejected from the orifices.^33^

Within 30 s and using just 1 mL of solution, many fibers can be gathered in a short space of time. PG spinning is a very rapid process, using small volumes means fast sample ejection. The time of 30 s was determined through previous experiments of examining the inside of the chamber postspinning to assess full solution ejection. In this work, 1 mL of solution was loaded for each sample to allow for fiber drying on contact with heat. It was found that smaller volumes allow for optimal fiber collection because of the aqueous nature of the solution. Greater control over humidity would likely enable larger volumes of fibers to be collected. In the PG spinning process, the spinning solution is distributed around the drum while it gathers speed on application of the motor, as such solution was ejected from all the orifices as the drum rotated. This generation of silk fibers using PG is significant because of the sheer quantity of that can be generated in a short period of time, combined with the absence of cytotoxic solvents. PG is capable of producing fibers orders of magnitude higher than ES, at a rate of up to 6 kg h^–1^ compared with only 0.17 kg h^–1^ and thus represents a significant step forward in green manufacturing of silk-based tissue engineering constructs.^29^

The PG spinning system recapitulates the silkworm’s natural silk spinning process to aid fiber formation. *B. mori* silk is a semicrystalline biopolymer that behaves as a liquid crystal when in solution and comprises ß-sheet crystals in a less ordered continuous phase.^34^ Natural silk fiber generation within the silk gland is a protein self-assembly process that involves forcing high concentrations of silk solution (25–50 w/v%) through a tapered spinning duct. Using PG, the spinning process applies shearing forces to the SF-based solutions that lead to chain entanglement and fiber formation as the proteins undergo phase transition and self-assemble into semicrystalline fibers.^17^ The robust mechanical properties of SF arise from strong hydrogen bonding that occurs among the ß-sheets when fibers are formed.^35^

### 3.2 Blend Solution Rheology with Respect to Fiber Formation

As with all fiber spinning processes, the solution properties, such as polymer concentration and molecular weight, solvent type, surface tension, and viscosity, have a marked effect on fiber formation.^36^ Differences in rotational viscosity values for each of the spinning solutions provides insight into their spinnability, as do surface tension values (Table 2). All measurements were performed in a range of 22.1–24 °C. A solution possessing surface tension and viscosity characteristics that are too low will not provide enough chain entanglement for fiber formation. Similarly, a solution that has too high of a viscosity or surface tension will not lead to fiber formation.

PEO is a common additive used to facilitate the SF fiber spinning.^37^ Previous work by Jin et al. demonstrated that adding PEO to silk solutions generated a viscosity and surface tension suitable for electrospinning.^28^ In this work, the addition of PEO to silk solutions was necessary to generate a viscosity and surface tension suitable for spinning, as even at ∼50 w/v% this is still insufficient. It is a biocompatible, water-based nontoxic filler that serves to improve the spinnability and enhances the elasticity of fibers, in addition to being water soluble.

A concentration of 15 w/v% was chosen because of its suitable performance in preliminary spinning experiments using PEO alone. Small additions of PEO at ratios of 80:20 and 90:10 have a significant effect on spinnability as entanglement density, and therefore viscosity, is increased. These findings are consistent with the work of Zhang et al., who created a dry spinning system using aqueous SF and graphene oxide, describing the purpose of the graphene oxide being to serve as a binder, facilitating chain entanglement via polar–polar and hydrophobic–hydrophobic interactions between the beta sheets of the SF.^38^

In the case of SF-HFIP, a polymer solution is formed through dissolution in the organic solvent that leads to formation of SF microfibrils.^34^ The solution behaves as a nematic liquid crystal and when shearing forces are then applied to the microfibrils via PG, this leads to ß-sheet crystal aggregation and fiber formation, as reported in our previous work.^27^ In the case of SF and HFIP the interaction between SF and HFIP in solution is different to that of SF and water. HFIP provides strong intermolecular forces to stabilize SF and create chain entanglement during the spinning process, as well as being highly volatile, allowing for fast evaporation and fiber formation.

Pure aqueous SF solution was unable to form fibers without the addition of PEO to the spinning system. The authors have attempted to spin at aqueous SF concentrations as high as 70 w/v% and no fiber formation was observed. This can be attributed to the lack of intermolecular interactions that occur at lower viscosities in the solution. For example, viscosity of SF-Aq was 19.3 ± 0.2 cP compared to 26.1 ± 0.2 cP of SF-PEO 90:10 (Table 2), the addition of PEO has a marked effect on solution parameters even in small quantities.

One explanation for the improved spinnability could be the effect of the addition of PEO on polydispersity of the regenerated silk fibroin solution. Palangetic et al. noted that extensional viscosity of a solution is a key factor in determining the stability of a filament during electrospinning. They noted that highly polydisperse systems lead to a reduction of the minimum required concentration for successful fiber formation, when compared to narrowly distributed polymer solutions of similar weight average molecular weights.^39^

The authors noted a linear relationship between shear stress and shear rate when recording rotational viscosity, for all samples of PEO-Aq, SF-PEO blends but not SF-Aq, indicating non-Newtonian behavior in the aqueous SF sample. Future analysis using cone–plate geometry over a wider range of shear rates would elucidate shear thinning behavior.

### 3.3 Effect of Spinning Parameters on Fiber Diameter and Distribution

The morphology and diameters of the PG-spun fibers were examined using SEM. As previously stated, pure SF-aq did not form fibers, and as such, the SEM images represent the remaining solutions. Multiple images were taken of each sample and fiber diameter analysis was performed using *ImageJ* software (*n* = 100). The fibers produced in this work range from 0.56–2.81 μm. The mean fiber diameters for varying spinning parameters are recorded in Table 3.

**Table 3:** Mean Diameters for Fibers Spun at a Range of Concentrations and Blends

	mean fiber diameter (μm)	SD	median (μm)	Shapiro–Wilk statistic	*P*-value
PEO-Aq					
0	0.59^a^	0.13	0.59	0.98	0.27
0.1	0.56^a^	0.12	0.56	0.98	0.09
0.2	0.73^a^	0.24	0.71	0.99	0.66
0.3	0.65	0.16	0.62	0.94	1.62 × 10^–4^
SF-PEO 80:20					
0	1.22	0.51	1.18	0.92	7.76 × 10^–6^
0.1	1.31^a^	0.4	1.25	0.98	0.14
0.2	1.46^a^	0.47	1.41	0.978	6.44 × 10^–2^
0.3	2.1^a^	0.78	1.99	0.98	0.06
SF-PEO 90:10					
0	0.71^a^	0.17	0.68	0.98	0.10
0.1	0.77^a^	0.22	0.76	0.98	0.08
0.2	1.63	0.49	1.56	0.97	0.03
0.3	1.27	0.44	1.21	0.92	1.49 × 10^–5^
SF-HFIP					
0	0.84	0.25	0.84	0.96	0.00
0.1	1.36	0.32	1.36	0.97	0.05
0.2	1.42	0.5	1.33	0.95	0.00
0.3	2.81	0.93	2.72	0.94	9.75 × 10^–5^

a Denotes likely normal result at 5%.

Pure PEO fibers yielded the smallest fiber diameters, although this was not statistically significant in 0 and 0.1 MPa samples. The highest fiber diameters were seen at 0.3 MPa pressure for the SF-HFIP and SF-PEO samples. The fiber diameters for the blended fibers ranged from 1.22–2.10 μm in 80:20 fibers, increasing with applied pressure. For the 90:10 fibers, diameters ranged from 0.71–1.63 μm.

In this work, structures exhibiting both micrometer and nanoscale features provide both mechanical integrity and high surface area needed for the attachment and growth of cells. It is hypothesized that the SF-PEO blend microfibers obtained from the aqueous route, which are all below 5 μm, have the potential to form a hybrid micronanoscale scaffold.^40^

In PG, the fiber outcome can be attributed to both the solution properties (i.e., viscosity, surface tension, molecular weight) and the spinning conditions (i.e., speed, applied pressure, collection distance, humidity, and temperature). The overlap among error bars for fiber diameter can possibly be attributed to the application of heat via a heat gun to the PG fiber spinning area as well as the relative humidity. Fluctuations in direction of heat flow and relative humidity during the fiber spinning and drying process can lead to variation in fiber diameter. The heat gun was used to reduce the humidity and aid fiber drying, providing a greater interface between liquid and air. However, the random motion of the fibers induced by using the gun may have resulted in some fibers joining together during their forming. These issues can be addressed by using a controlled atmosphere and heat application, as well as optimizing the collection of fibers, which newer generations of the PG device are able to provide.

Figure 3 shows SEMs of each of the solutions spun over the range of applied pressures. The SF-HFIP fibers were pore-free, beaded, and flat and ribbonlike in their morphology. This is consistent with our previous findings.^27^ The ribbonlike morphology often occurs due to increased mass transport as the solution is ejected from the orifices, and the rapid rate of HFIP evaporation, because of its volatility, causes the fiber to collapse into itself. All the fibers produced using SF-PEO blends are pore-free, and some branching is observed. PEO-Aq fibers do appear to be irregular in their diameter, which can be attributed to degradation of the PEO in atmosphere, demonstrating a significant use for blending with RSF.

**Figure 2:**
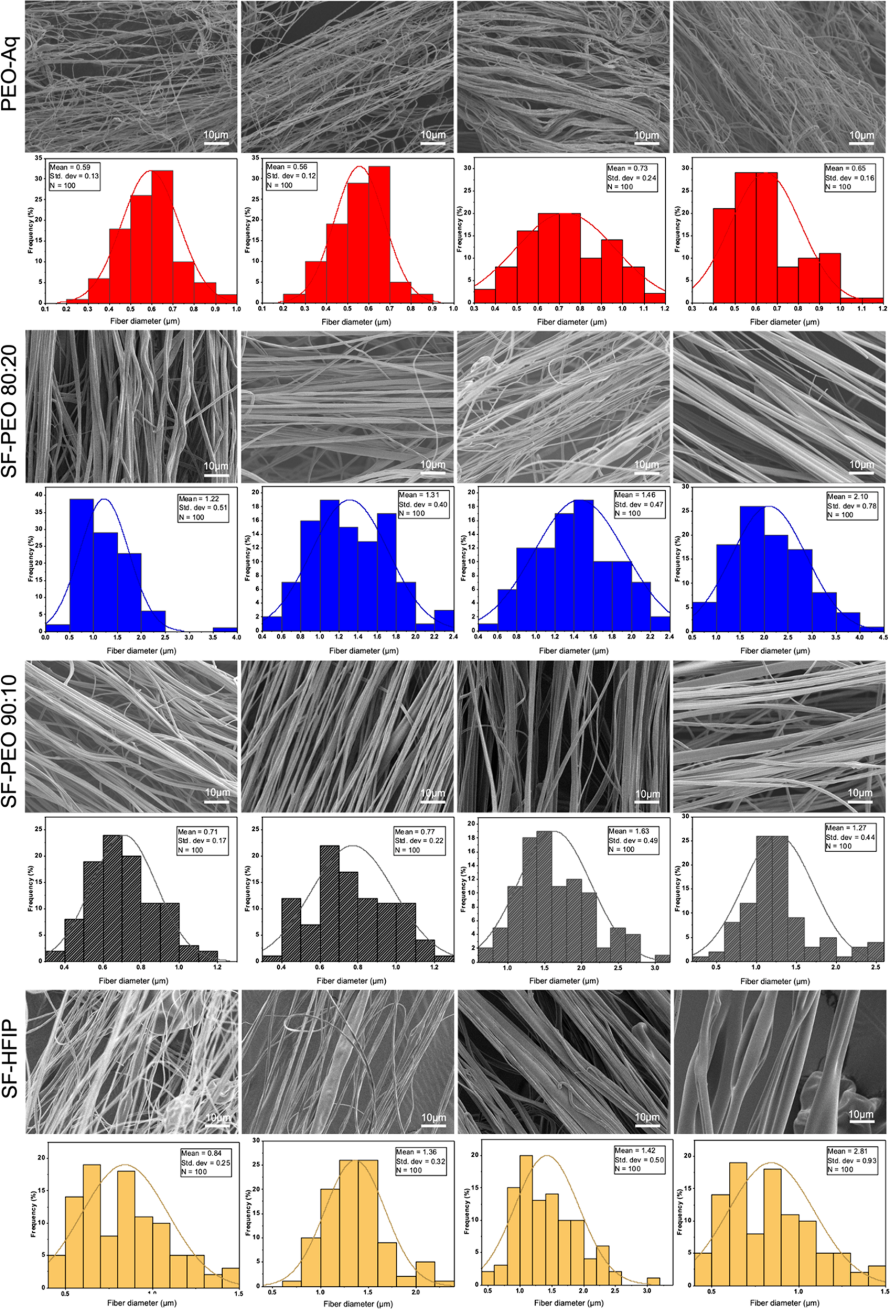
Scanning electron micrographs of fibers produced from different polymer blends at a range of applied pressures, all at a rotational speed of 36 000 rpm displayed with their corresponding histogram distribution plots.

All the fibers produced in this work are aligned, demonstrating the effectiveness of PG to produce large bundles of oriented fibers. Fiber alignment in scaffolds is considered to be beneficial in providing cues for the generation of oriented tissue structures that facilitate phenotypic differentiation of cell types, that may pose useful for the application of this research to bone tissue engineering.^41^ Chen et al. produced dexamethasone-loaded SF:PEO nanofibers via electrospinning for application to endothelial cell inflammation. Fiber mats were reported to be in the nanoscale although not aligned. ES is difficult to produce aligned fibers as they are spun in a random motion, although some groups have managed the preparation of aligned fibers through methods such as stable jet electrospinning.^42,43^ A normal distribution of fiber diameter is observed for PEO-Aq 0–0.2 MPa applied pressure, SF-PEO 80:20 0.1–0.3 MPa, SF-PEO 90:10 0–0.1 MPa, as indicated by the Shapiro–Wilk test in Table 3 and the fiber diameter distribution plots in Figure 2.

### 3.4 Attenuated Total Reflectance-Fourier Transform Infrared Spectroscopy (ATR-FTIR)

**Figure 3:**
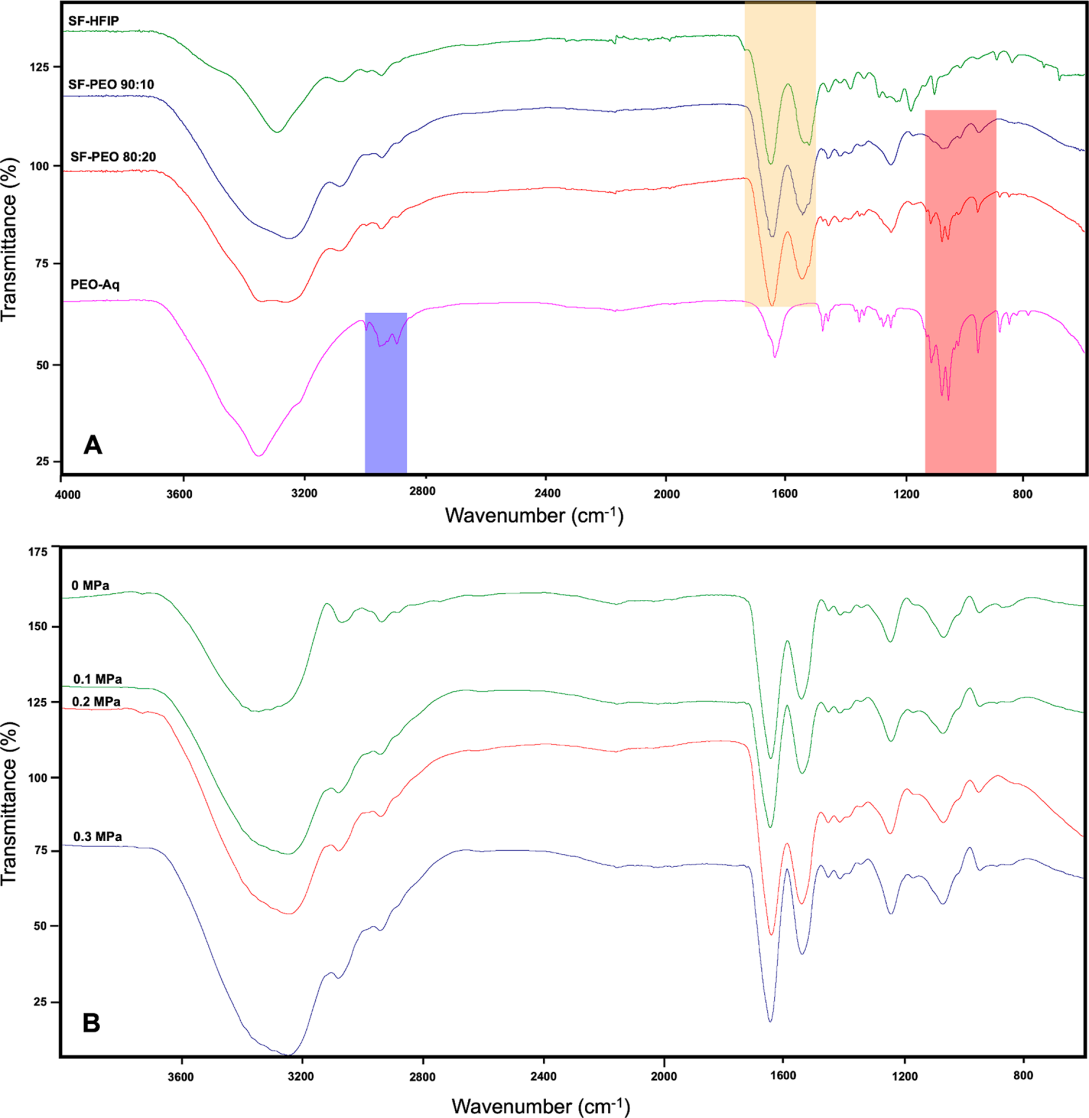
FTIR spectra of: (A) SF-HFIP, SF-PEO 80:20, SF-PEO 90:10, and PEO-Aq samples, (B) SF-PEO 90:10 samples obtained with varying pressures.

To investigate the characteristic absorption bands of SF and PEO, we obtained FTIR spectra of SF-HFIP, SF-PEO 80:20, SF-PEO 90:10, and PEO samples (Figure 3A). SF absorption bands reveal themselves at 1700–1600 cm^–1^ and 1600–1500 cm^–1^ as amide I and amide II, respectively, due to the secondary structure of the fibroin protein (yellow region). These can be attributed to C=O stretching vibration with an addition of N–H in-plane bending. The pristine PEO has primary characteristic absorption bands at 1100–1000 cm^–1^ due to C–O stretching and multiple weak absorptions at 3000–2900 cm^–1^ due to CH_2_ stretching (blue region). Notably, the C–O stretching peak diminishes gradually as the PEO ratio decreases in the blend (red region). PEO also has strong bands at 3300 and 1630 cm^–1^, indicating the strong hydroxyl and carbon-oxide vibrations, respectively. It is plausible that the presence of strong hydroxyl groups may be attributed to the absorption of moisture from air in the samples, as PEO is highly hydrophilic.

Figure 3B shows the infrared spectra of SF-PEO 90:10 samples obtained with varying pressures. The identical spectra indicate that the applied pressure has no effect on infrared absorption and chemical structure.

### 3.5 Thermogravimetric Analysis (TGA)

**Figure 4:**
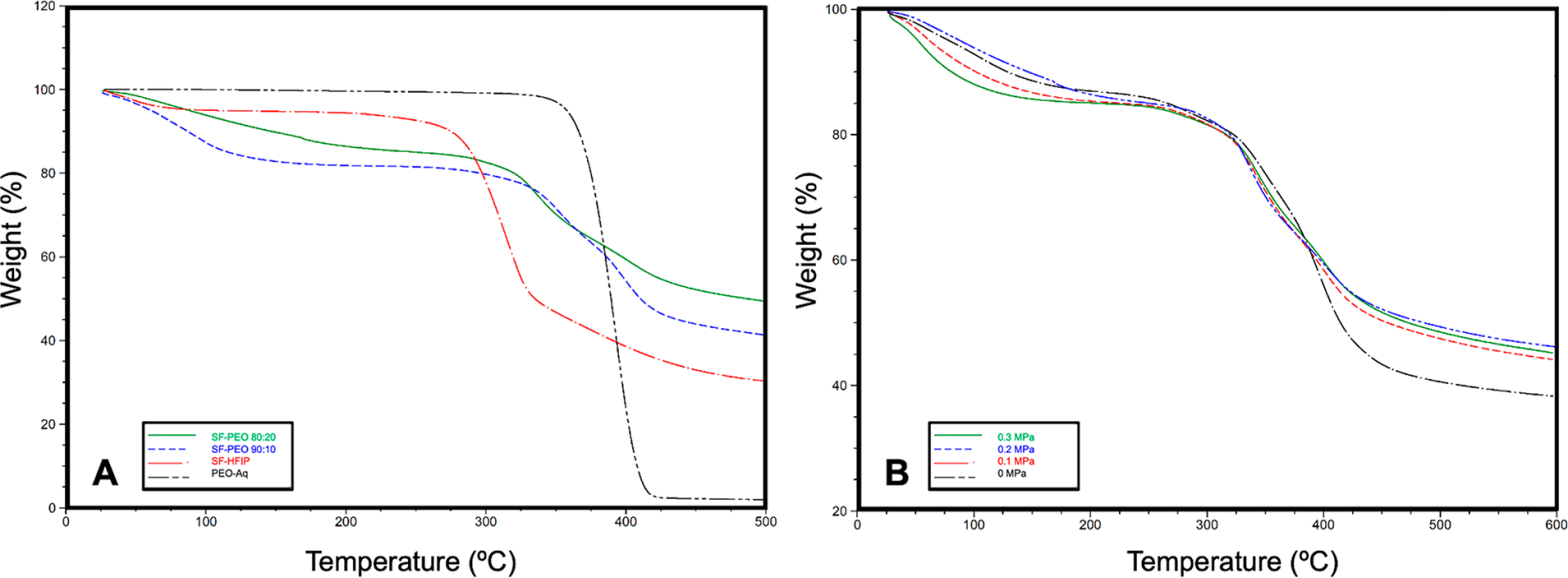
Thermal decomposition curves of (A) SF in HFIP, SF-PEO 80:20, SF-PEO 90:10, and PEO-Aq gyrospun fibers obtained with the same applied pressure, (B) SF-PEO 90:10 samples obtained with varying pressures.

Figure 4A shows the thermal decomposition curves of SF-HFIP, SF-PEO 80:20, SF-PEO 90:10, and PEO-Aq gyrospun fibers obtained with the same applied pressure. The figure indicates the enhanced thermal stabilities of the blend structure owing to strong interactions between the polymers. In both ratios of the polymer blend fibers, a multitype mass loss motive was observed. In the SF-HFIP sample, the structure lost its water content at approximately 90 °C and kept its thermal stability until 275 °C. It starts to decompose rapidly until 320 °C is reached, retaining 50% of its mass with a slow decomposition rate.

Meanwhile, pristine PEO completely decomposed at 380–410 °C temperature range. Although the SF component of the blended structure begins to degrade at lower temperatures, the amount of incorporated PEO helps the structure to keep its mass up to more than 30% than the pure SF sample (up to 380 °C), where its thermal degradation begins. This result originates from the strong intermolecular hydrogen bonding between the two polymer types, which enhances the thermal stability of the fibers. The thermal decomposition curves of SF-PEO 90:10 samples obtained with varying pressures exhibit a similar multistep degradation profile, indicating that the applied pressure has no significant effect on thermal stability (Figure 4B).

### 3.6 Cytotoxicity Evaluation

**Figure 5:**
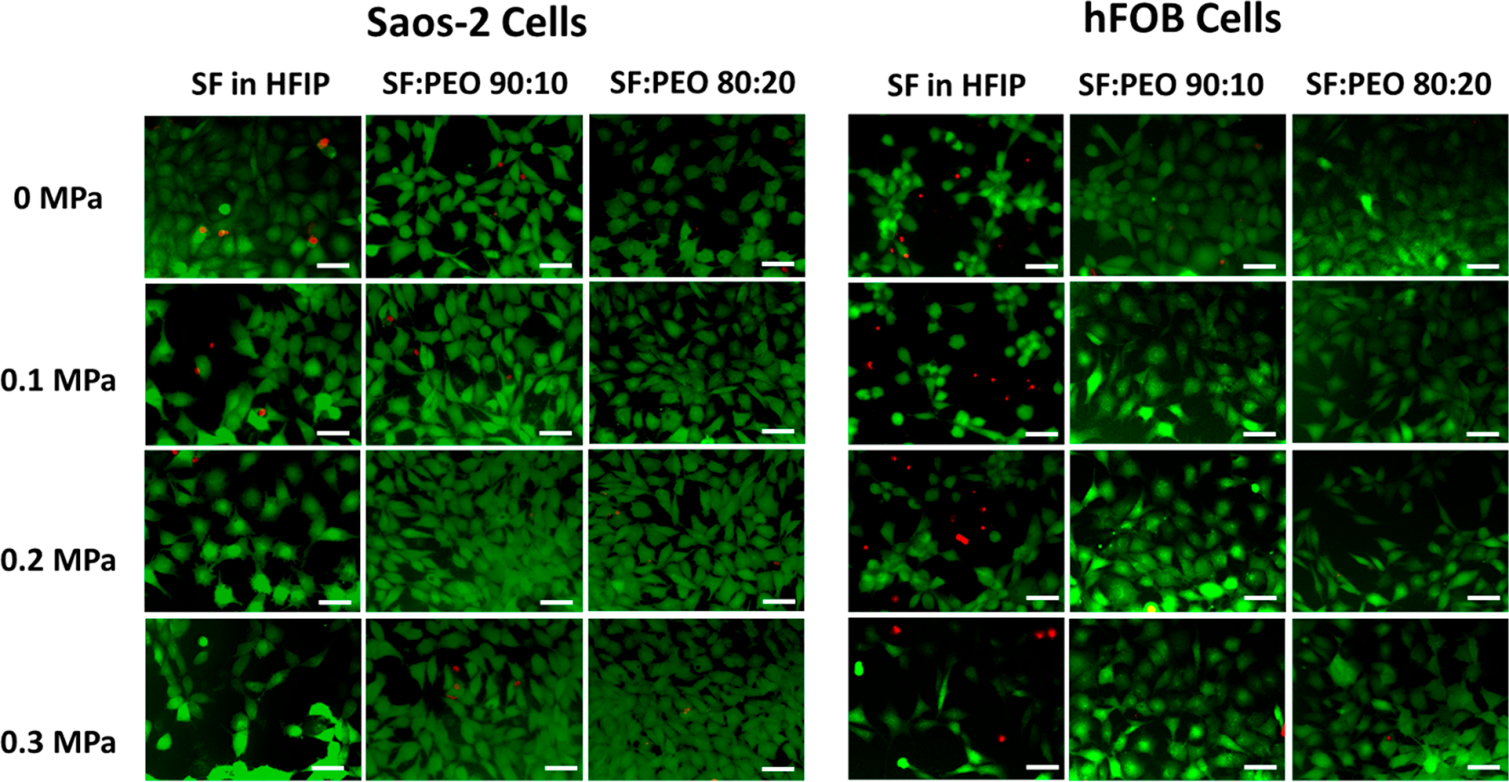
Live/Dead cell viability assay in SaOS-2 and hFob cells: representative fluorescence microscopy images of data obtained from each sample group in a single day, where green fluorescence indicates live and red fluorescence signals indicate dead cells (scale bar = 100 μm).

Figure 5 shows representative Live/Dead assay fluorescence microscopy images of each sample group, where green fluorescence indicates live cells, and red fluorescence indicates dead cells, due to DNA staining. SF-HFIP samples demonstrated a greater presence of red fluorescence than the other sample groups. This result also correlates with the MTT assay data, where percentage viability of the SF-HFIP samples was lower than blended samples. Additionally, cell densities were found to be higher in blended sample groups, whereas number of adhered cells in each representative image were lower in the SF-HFIP group.

The cell proliferation rate on gyrospun cells was evaluated directly by seeding each cell type onto gyrospun fibers and measuring using the MTT assay. Viable cells metabolize the MTT reagent in their mitochondria and form dark blue formazan crystals, which can be further dissolved to evaluate percentage viability among sample groups through optical density.

**Figure 6:**
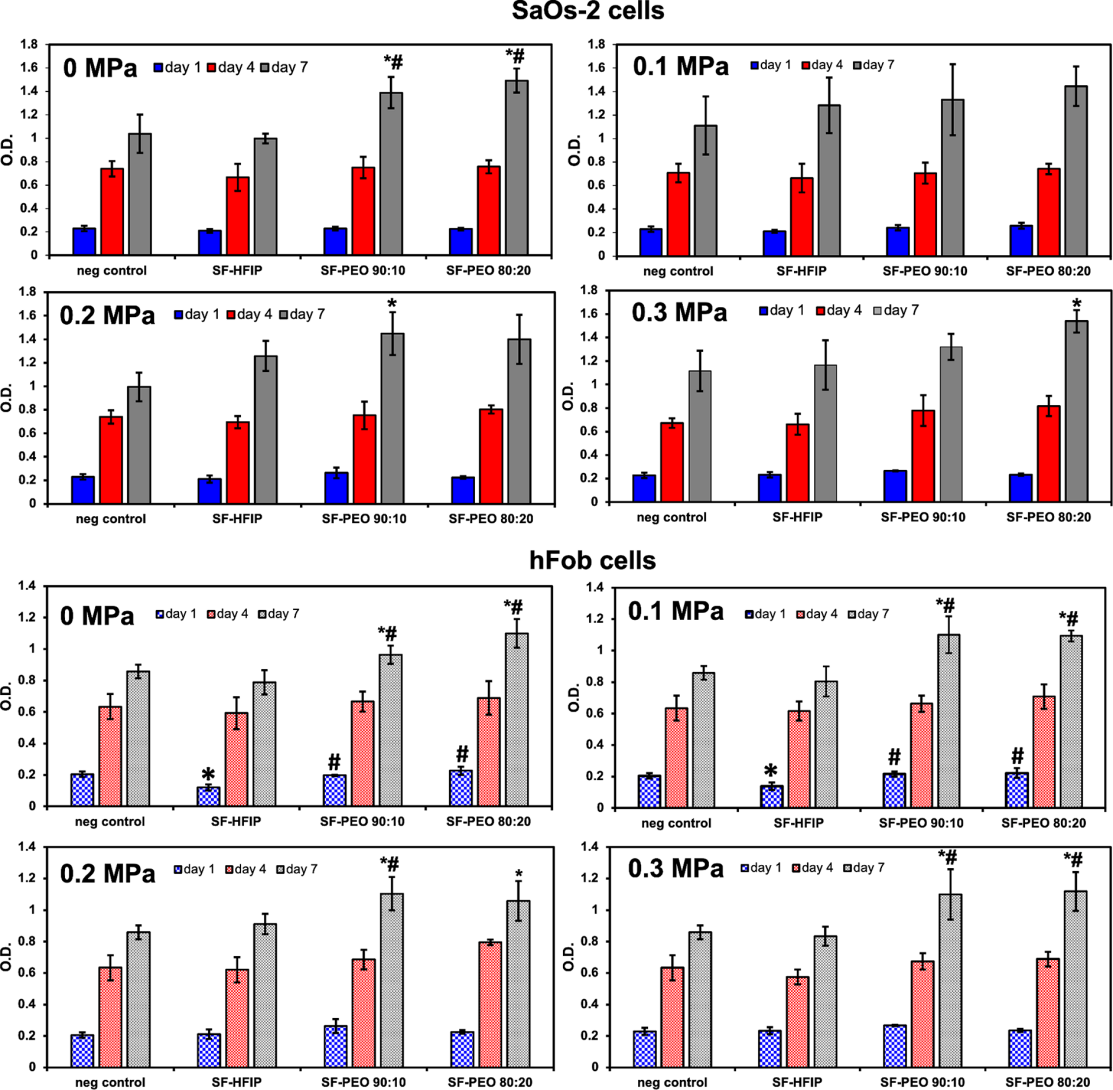
Cell proliferation assay using an MTT test. * and # symbols on top of the columns denote statistical significance with respect to other related groups in each graph; * shows the significant difference of the group with respect to the negative control and # shows the significance with respect to SF in the HFIP sample.

Figure 6 shows the formazan absorption of SaOS-2 cells cultured on gyrospun fibers for 1, 4, and 7 days. The number of viable SaOS-2 cells increased with incubation time for all sample groups, including all blended ratios and applied pressures. The number of cells in all sample groups were enhanced due to increased surface area against the bottom of the tissue culture plate, and no evidence of cytotoxicity was observed. In addition to the absence of any cytotoxic effect, the proliferation rate was found to be higher because the three-dimensional organization of the fibers provides more surface area for the cells. The number of cells on the seventh day was found to be higher in SF-PEO blended samples in all varying pressures, however this increase was statistically significant only in samples obtained without the application of pressure. A one-way ANOVA test confirmed that the only statistical significance occurred among the sample groups at day 7 for 0, 0.2, and 0.3 MPa pressures. For 0 MPa, both 80:20 and 90:10 blends are statistically significantly higher than both negative control and SF-HFIP samples. For 0.1 MPa, no statistical significance was observed among groups per day. For 0.2 MPa, 90:10 is significantly higher than the negative control and for 0.3 MPa, 80:20 is significantly higher than the negative control.

On the other hand, hFob cell proliferation was found to be significantly higher in blended fibers than the control and SF-HFIP sample groups on day 4 and day 7. In addition to the osteosarcoma test group, on day one, there were significantly fewer numbers of cells in the SF-HFIP group than all three samples, when the applied pressure was less than 0.2 MPa. This finding could possibly be attributed to the incomplete evaporation of HFIP during the gyration process at low pressures.

The increased cell proliferation rates in osteoblast cell lines demonstrate the significance of this work for bone tissue engineering. The use of SF fibers in bone tissue engineering is well documented, as it is a flexible, highly biocompatible polymer that can be tailored to include the addition of hydroxyapatite and other molecules that enhance bone regeneration.^44^ ES of fibers for bone tissue engineering has been hampered by reproducibility issues with the method because of the difficulty in controlling the many parameters that affect fiber formation.^45^ The gyratory forming method described here allows for rapid generation of fibers, yet more work is needed to elucidate control over formation and morphology. The use of the heat gun in the formation that is described in this work is limiting as it adds another parameter that must be controlled. Work is underway in our group to automate the pressurized gyration system and induce control on factors such as temperature and humidity, which in turn will allow for greater control over solution blowing as the fibers are ejected from the orifices of the drum. As SF is significant in its effect on bone formation, future work will involve more detailed assessment and refinement of the mechanical properties of the fibers produced using this aqueous spinning system.

## Conclusions

The work described here represents a promising progressive step in the scaling of green chemistry production of SF fibers. PG was shown as a suitable method to rapidly produce fibers from an aqueous spinning system. Microscale fibers were achieved ranging from 0.71–2.10 μm. To date, the authors do not know of another method that has been able to rapidly generate large quantities of aligned aqueous-based RSF fibers. The fibers were shown to support significant cell viability and proliferation in an osteosarcoma SaOS-2 cell line and in human fetal osteoblast (hFob) cells when compared to fibers produced using HFIP. Future studies will involve further refinement of the PG setup to enhance fiber reproducibility and investigating the mechanical properties of fibers required for bone tissue engineering. In vitro and possibly in vivo degradation studies of gyrospun fibers are needed in the future for a comprehensive evaluation of the spun fibers. However, in this very first step of our “synthesis of silk fibroin via aqueous route” investigation, we focused more on physicochemical analyses and cytotoxicity evaluations.

## Supporting Information Available

Video S1, generation of SF-PEO 90:10 fibers in the pressurized gyration chamber following 30 s of spinning (MP4)

## References

[ref1] Tandon S.; Kandasubramanian B.; Ibrahim S. M. Silk-Based Composite Scaffolds for Tissue Engineering Applications. Ind. Eng. Chem. Res. 2020, 59 (40), 17593–17611. 10.1021/acs.iecr.0c02195.

[ref2] Bae S. B.; Kim M. H.; Park W. H. Electrospinning and Dual Crosslinking of Water-Soluble Silk Fibroin Modified with Glycidyl Methacrylate. Polym. Degrad. Stab. 2020, 179, 109304 10.1016/j.polymdegradstab.2020.109304.

[ref3] Terkaj W.; Tolio T. The Italian Flagship Project: Factories of the Future. In Factories of the Future: The Italian Flagship Initiative; Terkaj W., Tolio T., Copani G., Eds.; Springer, 2019; pp 3–35. 10.1007/978-3-319-94358-9_1

[ref4] Orash Mahmoud Salehi A.; Nourbakhsh M. S.; Rafienia M.; Baradaran-Rafii A.; Heidari Keshel S. Corneal Stromal Regeneration by Hybrid Oriented Poly (ε-Caprolactone)/Lyophilized Silk Fibroin Electrospun Scaffold. Int. J. Biol. Macromol. 2020, 161, 377 10.1016/j.ijbiomac.2020.06.045. 32526297

[ref5] Varshney N.; Sahi A. K.; Poddar S.; Mahto S. K. Soy Protein Isolate Supplemented Silk Fibroin Nanofibers for Skin Tissue Regeneration: Fabrication and Characterization. Int. J. Biol. Macromol. 2020, 160, 112 10.1016/j.ijbiomac.2020.05.090. 32422270

[ref6] Maharjan B.; Kaliannagounder V. K.; Jang S. R.; Awasthi G. P.; Bhattarai D. P.; Choukrani G.; Park C. H.; Kim C. S. In-Situ Polymerized Polypyrrole Nanoparticles Immobilized Poly(ε-Caprolactone) Electrospun Conductive Scaffolds for Bone Tissue Engineering. Mater. Sci. Eng., C 2020, 114, 111056 10.1016/j.msec.2020.111056. 32994008

[ref7] Zhao G.; Zhang X.; Li B.; Huang G.; Xu F.; Zhang X. Solvent-Free Fabrication of Carbon Nanotube/Silk Fibroin Electrospun Matrices for Enhancing Cardiomyocyte Functionalities. ACS Biomater. Sci. Eng. 2020, 6 (3), 1630–1640. 10.1021/acsbiomaterials.9b01682. 33455382

[ref8] Xu X.; Ren S.; Li L.; Zhou Y.; Peng W.; Xu Y. Biodegradable Engineered Fiber Scaffolds Fabricated by Electrospinning for Periodontal Tissue Regeneration. J. Biomater. Appl. 2021, 36 (1), 55–75. 10.1177/0885328220952250. 32842852

[ref9] Xiao L.; Lu G.; Lu Q.; Kaplan D. L. Direct Formation of Silk Nanoparticles for Drug Delivery. ACS Biomater. Sci. Eng. 2016, 2 (11), 2050–2057. 10.1021/acsbiomaterials.6b00457. 33440541

[ref10] Koh L. D.; Yeo J.; Lee Y. Y.; Ong Q.; Han M.; Tee B. C. K. Advancing the Frontiers of Silk Fibroin Protein-Based Materials for Futuristic Electronics and Clinical Wound-Healing (Invited Review). Mater. Sci. Eng., C 2018, 86, 151–172. 10.1016/j.msec.2018.01.007. 29525090

[ref11] Patil A. C.; Bandla A.; Liu Y. H.; Luo B.; Thakor N. V. Nontransient Silk Sandwich for Soft, Conformal Bionic Links. Mater. Today 2020, 32, 68–83. 10.1016/j.mattod.2019.08.007.

[ref12] Cho H. J.; Ki C. S.; Oh H.; Lee K. H.; Um I. C. Molecular Weight Distribution and Solution Properties of Silk Fibroins with Different Dissolution Conditions. Int. J. Biol. Macromol. 2012, 51 (3), 336–341. 10.1016/j.ijbiomac.2012.06.007. 22705473

[ref13] Wang L.; Luo Z.; Zhang Q.; Guan Y.; Cai J.; You R.; Li X. Effect of Degumming Methods on the Degradation Behavior of Silk Fibroin Biomaterials. Fibers Polym. 2019, 20 (1), 45–50. 10.1007/s12221-019-8658-9.

[ref14] Wray L. S.; Hu X.; Gallego J.; Georgakoudi I.; Omenetto F. G.; Schmidt D.; Kaplan D. L. Effect of Processing on Silk-Based Biomaterials: Reproducibility and Biocompatibility. J. Biomed. Mater. Res. - Part B Appl. Biomater. 2011, 99 (1), 89–101. 10.1002/jbm.b.31875. PMC3418605 21695778

[ref15] Holland C.; Numata K.; Rnjak-Kovacina J.; Seib F. P. The Biomedical Use of Silk: Past, Present, Future. Adv. Healthc. Mater. 2019, 8 (1), 1800465 10.1002/adhm.201800465. 30238637

[ref16] Jahangirian H.; Ghasemian Lemraski E.; Rafiee-Moghaddam R.; Webster T. A Review of Using Green Chemistry Methods for Biomaterials in Tissue Engineering. Int. J. Nanomedicine 2018, 13, 5953–5969. 10.2147/IJN.S163399. 30323585 PMC6177385

[ref17] Guo C.; Li C.; Mu X.; Kaplan D. L. Engineering Silk Materials: From Natural Spinning to Artificial Processing. Appl. Phys. Rev. 2020, 7 (1), 011313 10.1063/1.5091442. 34367402 PMC8340942

[ref18] Li X.; Ming J.; Ning X. Wet-Spun Conductive Silk Fibroin–Polyaniline Filaments Prepared from a Formic Acid–Shell Solution. J. Appl. Polym. Sci. 2019, 136, 47127 10.1002/app.47127.

[ref19] Nalvuran H.; Elçin A. E.; Elçin Y. M. Nanofibrous Silk Fibroin/Reduced Graphene Oxide Scaffolds for Tissue Engineering and Cell Culture Applications. Int. J. Biol. Macromol. 2018, 114, 77–84. 10.1016/j.ijbiomac.2018.03.072. 29551508

[ref20] Lim S. H.; Mao H. Q. Electrospun Scaffolds for Stem Cell Engineering. Adv. Drug Delivery Rev. 2009, 61, 1084 10.1016/j.addr.2009.07.011. 19647024

[ref21] Kishimoto Y.; Morikawa H.; Yamanaka S.; Tamada Y. Electrospinning of Silk Fibroin from All Aqueous Solution at Low Concentration. Mater. Sci. Eng., C 2017, 73, 498–506. 10.1016/j.msec.2016.12.113. 28183638

[ref22] Doostmohammadi M.; Forootanfar H.; Ramakrishna S. Regenerative Medicine and Drug Delivery: Progress via Electrospun Biomaterials. Mater. Sci. Eng., C 2020, 109, 110521 10.1016/j.msec.2019.110521. 32228899

[ref23] Wang H.; Shao H.; Hu X. Structure of Silk Fibroin Fibers Made by an Electrospinning Process from a Silk Fibroin Aqueous Solution. J. Appl. Polym. Sci. 2006, 101 (2), 961–968. 10.1002/app.24024.

[ref24] Liu C.; Sun J.; Shao M.; Yang B. A Comparison of Centrifugally-Spun and Electrospun Regenerated Silk Fibroin Nanofiber Structures and Properties. RSC Adv. 2015, 5 (119), 98553–98558. 10.1039/C5RA15486C.

[ref25] Jin Y.; Zhang Y.; Hang Y.; Shao H.; Hu X. A Simple Process for Dry Spinning of Regenerated Silk Fibroin Aqueous Solution. J. Mater. Res. 2013, 28 (20), 2897–2902. 10.1557/jmr.2013.276.

[ref26] Luo J.; Zhang L.; Peng Q.; Sun M.; Zhang Y.; Shao H.; Hu X. Tough Silk Fibers Prepared in Air Using a Biomimetic Microfluidic Chip. Int. J. Biol. Macromol. 2014, 66, 319–324. 10.1016/j.ijbiomac.2014.02.049. 24613677

[ref27] Heseltine P. L.; Hosken J.; Agboh C.; Farrar D.; Homer-Vanniasinkam S.; Edirisinghe M. Fiber Formation from Silk Fibroin Using Pressurized Gyration. Macromol. Mater. Eng. 2019, 304 (1), 1800577 10.1002/mame.201800577.

[ref28] Jin H.-J.; Fridrikh S. V.; Rutledge G. C.; Kaplan D. L. Electrospinning *Bombyx Mori* Silk with Poly(Ethylene Oxide). Biomacromolecules 2002, 3 (6), 1233–1239. 10.1021/bm025581u. 12425660

[ref29] Mahalingam S.; Edirisinghe M. Forming of Polymer Nanofibers by a Pressurised Gyration Process. Macromol. Rapid Commun. 2013, 34 (14), 1134–1139. 10.1002/marc.201300339. 23749758

[ref30] Wang X.; Rockwood D. N.; Kaplan D. L.; Yücel T.; Lovett M. L.; Preda R. C. Materials Fabrication from Bombyx Mori Silk Fibroin. Nat. Protoc. 2011, 6 (10), 1612–1631. 10.1038/nprot.2011.379. 21959241 PMC3808976

[ref31] Raimi-Abraham B. T.; Mahalingam S.; Davies P. J.; Edirisinghe M.; Craig D. Q. M. Development and Characterization of Amorphous Nanofiber Drug Dispersions Prepared Using Pressurized Gyration. Mol. Pharmaceutics 2015, 12 (11), 3851–3861. 10.1021/acs.molpharmaceut.5b00127. 26402331

[ref32] Raimi-Abraham B. T.; Mahalingam S.; Edirisinghe M.; Craig D. Q. M. Generation of Poly(N-Vinylpyrrolidone) Nanofibres Using Pressurised Gyration. Mater. Sci. Eng., C 2014, 39 (1), 168–176. 10.1016/j.msec.2014.02.016. 24863213

[ref33] Alenezi H.; Cam M. E.; Edirisinghe M. Experimental and Theoretical Investigation of the Fluid Behavior during Polymeric Fiber Formation with and without Pressure. Appl. Phys. Rev. 2019, 6 (4), 041401 10.1063/1.5110965.

[ref34] Ling S.; Qin Z.; Li C.; Huang W.; Kaplan D. L.; Buehler M. J. Polymorphic Regenerated Silk Fibers Assembled through Bioinspired Spinning. Nat. Commun. 2017, 8 (1), 1387 10.1038/s41467-017-00613-5. 29123097 PMC5680232

[ref35] Guo C.; Li C.; Vu H. V.; Hanna P.; Lechtig A.; Qiu Y.; Mu X.; Ling S.; Nazarian A.; Lin S. J.; Kaplan D. L. Thermoplastic Moulding of Regenerated Silk. Nat. Mater. 2020, 19 (1), 102–108. 10.1038/s41563-019-0560-8. 31844276 PMC6986341

[ref36] Amariei N.; Manea L. R.; Bertea A. P.; Bertea A.; Popa A. The Influence of Polymer Solution on the Properties of Electrospun 3D Nanostructures. IOP Conference Series: Materials Science and Engineering 2017, 209, 012092 10.1088/1757-899X/209/1/012092.

[ref37] Kopp A.; Smeets R.; Gosau M.; Kröger N.; Fuest S.; Köpf M.; Kruse M.; Krieger J.; Rutkowski R.; Henningsen A.; Burg S. Effect of Process Parameters on Additive-Free Electrospinning of Regenerated Silk Fibroin Nonwovens. Bioact. Mater. 2020, 5 (2), 241–252. 10.1016/j.bioactmat.2020.01.010. 32123778 PMC7036448

[ref38] Zhang C.; Zhang Y.; Shao H.; Hu X. Hybrid Silk Fibers Dry-Spun from Regenerated Silk Fibroin/Graphene Oxide Aqueous Solutions. ACS Appl. Mater. Interfaces 2016, 8 (5), 3349–3358. 10.1021/acsami.5b11245. 26784289

[ref39] Palangetic L.; Reddy N. K.; Srinivasan S.; Cohen R. E.; McKinley G. H.; Clasen C. Dispersity and Spinnability: Why Highly Polydisperse Polymer Solutions Are Desirable for Electrospinning. Polymer (Guildf) 2014, 55 (19), 4920–4931. 10.1016/j.polymer.2014.07.047.

[ref40] Naghashzargar E.; Farè S.; Catto V.; Bertoldi S.; Semnani D.; Karbasi S.; Tanzi M. C. Nano/Micro Hybrid Scaffold of PCL or P3Hb Nanofibers Combined with Silk Fibroin for Tendon and Ligament Tissue Engineering. J. Appl. Biomater. Funct. Mater. 2015, 13 (2), e156–e168. 10.5301/jabfm.5000216. 25589157

[ref41] Meinel A. J.; Kubow K. E.; Klotzsch E.; Garcia-Fuentes M.; Smith M. L.; Vogel V.; Merkle H. P.; Meinel L. Optimization Strategies for Electrospun Silk Fibroin Tissue Engineering Scaffolds. Biomaterials 2009, 30 (17), 3058–3067. 10.1016/j.biomaterials.2009.01.054. 19233463 PMC3792584

[ref42] Chen W.; Li D.; EI-Shanshory A.; El-Newehy M.; EI-Hamshary H. A.; Al-Deyab S. S.; He C.; Mo X. Dexamethasone Loaded Core-Shell SF/PEO Nanofibers via Green Electrospinning Reduced Endothelial Cells Inflammatory Damage. Colloids Surfaces B Biointerfaces 2015, 126, 561–568. 10.1016/j.colsurfb.2014.09.016. 25481687

[ref43] Yuan H.; Zhao S.; Tu H.; Li B.; Li Q.; Feng B.; Peng H.; Zhang Y. Stable Jet Electrospinning for Easy Fabrication of Aligned Ultrafine Fibers. J. Mater. Chem. 2012, 22 (37), 19634–19638. 10.1039/c2jm33728b.

[ref44] Nam J.; Huang Y.; Agarwal S.; Lannutti J. Materials Selection and Residual Solvent Retention in Biodegradable Electrospun Fibers. J. Appl. Polym. Sci. 2008, 107 (3), 1547–1554. 10.1002/app.27063.

[ref45] Liu H.; Xu G. W.; Wang Y. F.; Zhao H. S.; Xiong S.; Wu Y.; Heng B. C.; An C. R.; Zhu G. H.; Xie D. H. Composite Scaffolds of Nano-Hydroxyapatite and Silk Fibroin Enhance Mesenchymal Stem Cell-Based Bone Regeneration via the Interleukin 1 Alpha Autocrine/Paracrine Signaling Loop. Biomaterials 2015, 49 (0), 103–112. 10.1016/j.biomaterials.2015.01.017. 25725559

